# Supplementary figures and images for: Dynamics of tissue repair regulatory T cells and damage in acute Trypanosoma cruzi infection
Source: PLoS Pathog. 2025 Jan 30;21(1):e1012906. doi: 10.1371/journal.ppat.1012906 (PMC11813105; doi:10.1371/journal.ppat.1012906)

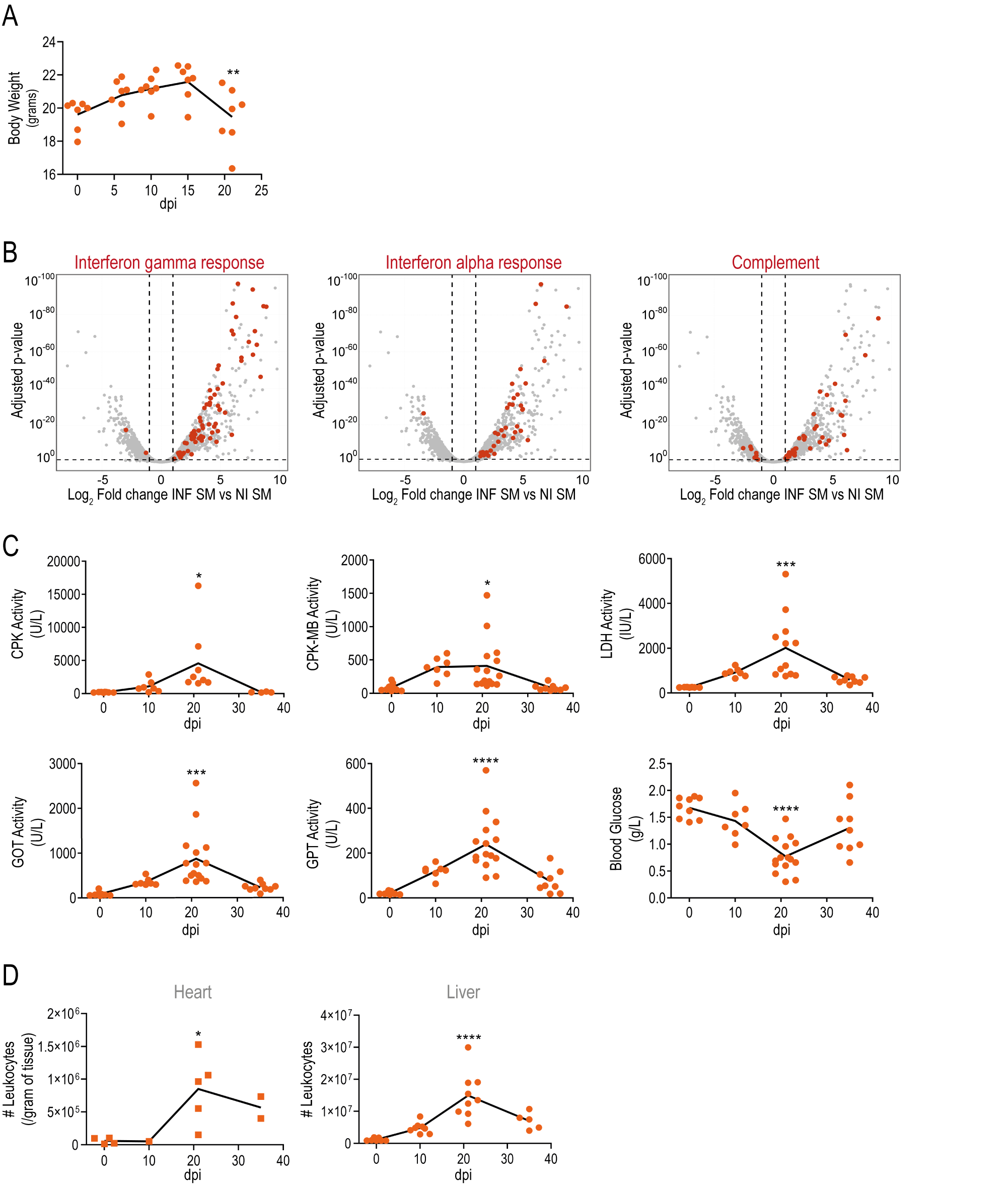

Supplement: S1 Fig — Indicators of disease progression and inflammatory response were evaluated in T. cruzi infected Foxp3-GFP mice at different days post infection (dpi). (A) Kinetics of total body weight (B) Whole quadriceps muscle (SM) RNAseq data analysis from non-infected (NI) and infected (INF) mice as described in Fig 1G and 1H; N = 3 per group. Volcano plots displaying differentially expressed genes (dots) between INF SM and NI SM. According to Fig 1G, genes associated with interferon gamma response, interferon alpha response and complement pathways are highlighted in red. (C) Kinetics of plasma CPK, CPK-MB, LDH, GOT and GPT activities, and glucose concentration. (D) Kinetics of leukocyte counts in heart normalized to tissue weight (left) and total leukocyte counts in liver (right). In (A and C) data are presented as individual values (circles) while the lines represent the mean. In (D), squares in heart represents values obtained from pools of 4–5 mice, while circles in liver represent values from individual mice, with the lines representing the mean. (A, C and D) Data were collected from 1–3 independent experiments. Statistical significance in (A) was determined by RM one-way ANOVA and P values are relative to 15 dpi, while in (B and C) was determined by one-way ANOVA and P values are relative to 0 dpi. *p < 0.05; **p<0.01; ****p < 0.0001. (TIF) [file ppat.1012906.s001.tif]

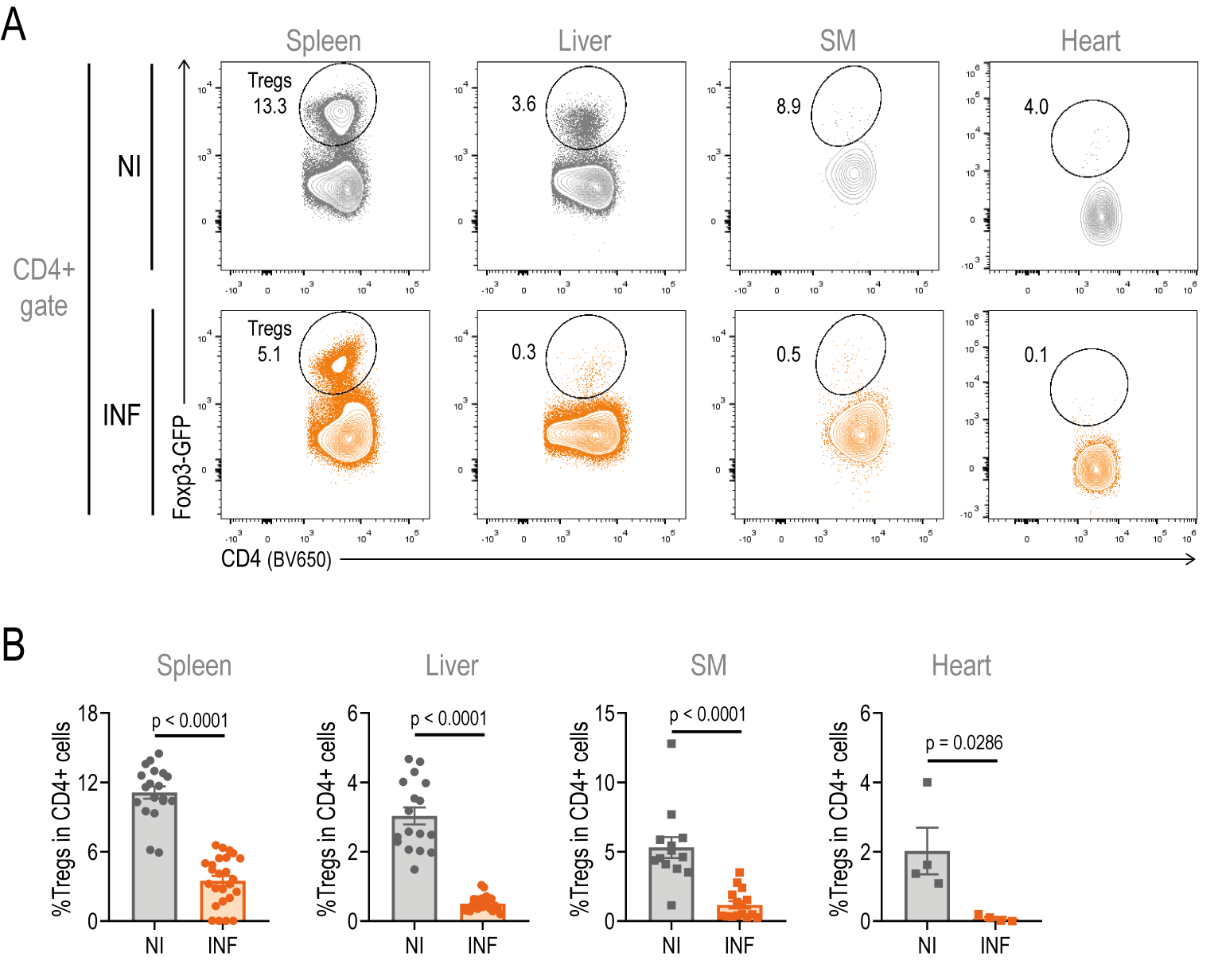

Supplement: S2 Fig — Tregs response was evaluated by flow cytometry in spleen, liver, skeletal muscle (SM) and heart from non-infected (NI) and infected (INF) (21 days post infection) Foxp3-GFP mice. (A) Representative dot plots showing the frequency of Tregs (CD4+ Foxp3-GFP+) within total CD4+ cells from each tissue. (B) Bars displaying Tregs frequency within total CD4+ cells as the mean ± SEM. Circles represent individual mice and squares represent pools of 3–5 mice. Statistical significance was determined by unpaired t test for spleen, liver and SM; and by Mann-Whitney test for heart. P values are indicated in the graphs. (A and B) Data were collected from 3 independent experiments. (TIF) [file ppat.1012906.s002.tif]

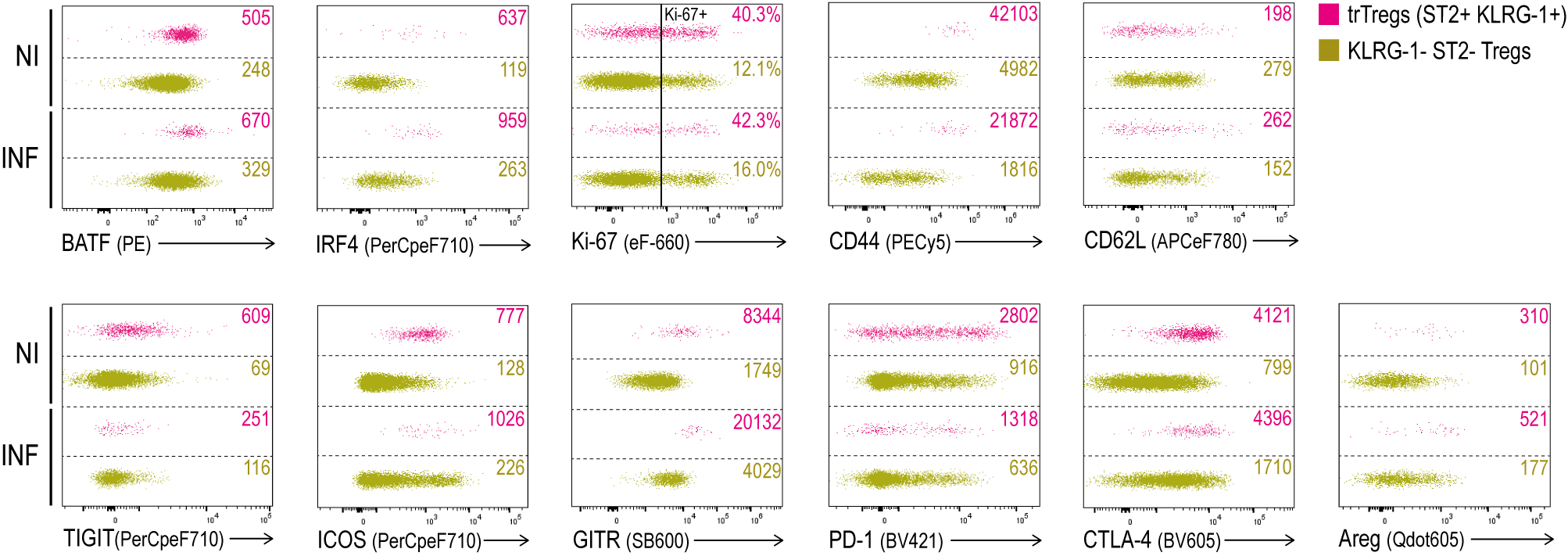

Supplement: S3 Fig — Flow cytometry phenotypic analysis of Tregs subsets present in the spleen of non-infected (NI) or infected (INF) (21 days post infection) Foxp3-GFP mice. Representative dot plots showing the expression of each cell marker in ST2+ KLRG-1+ (pink) and ST2- KLRG-1- (gold) Tregs as defined in Fig 2A. Numbers on top right corner of each plot indicate either mean fluorescence intensity or frequency of positive cells for each marker. Data were collected from 3 independent experiments. (TIF) [file ppat.1012906.s003.tif]

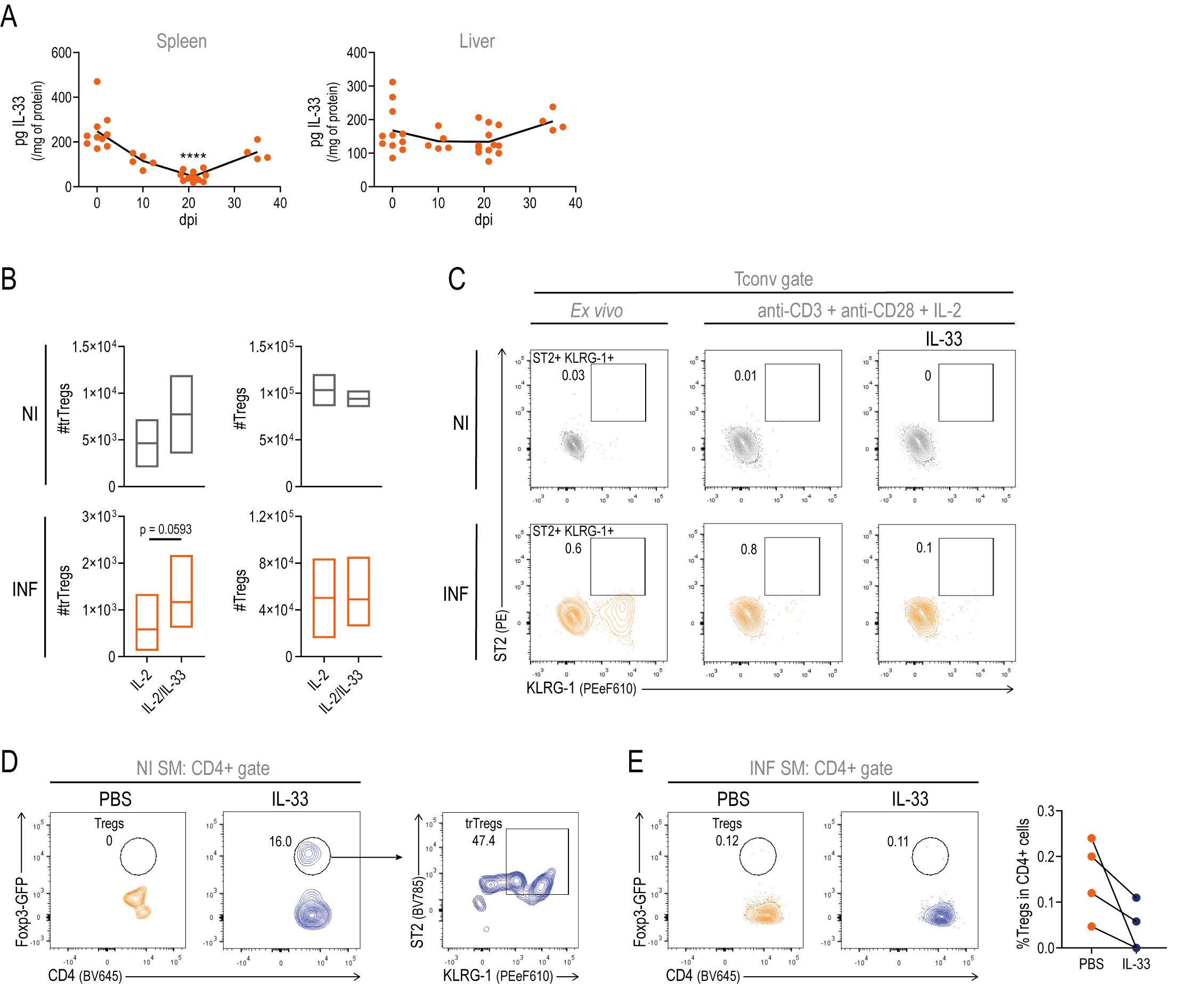

Supplement: S4 Fig — (A) IL-33 concentration was evaluated in spleen and liver lysates obtained from Foxp3-GFP mice at different days post infection (dpi). Values were normalized to total protein content. Data are presented as individual replicates (circles) and mean (line). (B) Floating bars represent the cumulative counts of live ST2+ KLRG-1+ Tregs (trTregs) and total Tregs after culturing splenic Tregs isolated from non-infected (NI) and infected (INF) Foxp3-GFP mice, as described in Fig 3B. Horizontal lines indicate the average value; N = 2–3 per group. (C) Foxp3-GFPneg CD4+ conventional T cells (Tconv) isolated from the spleen of NI and INF Foxp3-GFP mice were evaluated by flow cytometry. Representative dot plots showing ST2+ KLRG-1+ cells frequency within total Tconv. Left plots correspond to uncultured Tconv, while middle and right plots correspond to Tconv activated with anti-CD3+anti-CD23+IL-2 with or without IL-33 for 72 hours. N = 3 NI and 3 INF. (D and E) Representative plots depicting the frequencies of GFP+ CD4+ Tregs (D and E) and ST2+ KLRG-1+ cells (D) in NI (D) and INF (E) mice receiving intramuscular IL-33. Statistical significance was determined by Kruskal-Wallis test (A), paired t test (B) and Wilcoxon test (E). P values in (A) are relative to 0 dpi: ****p < 0.0001. Data are representative of two (A-C) and one (D and E) independent experiments. (TIF) [file ppat.1012906.s004.tif]

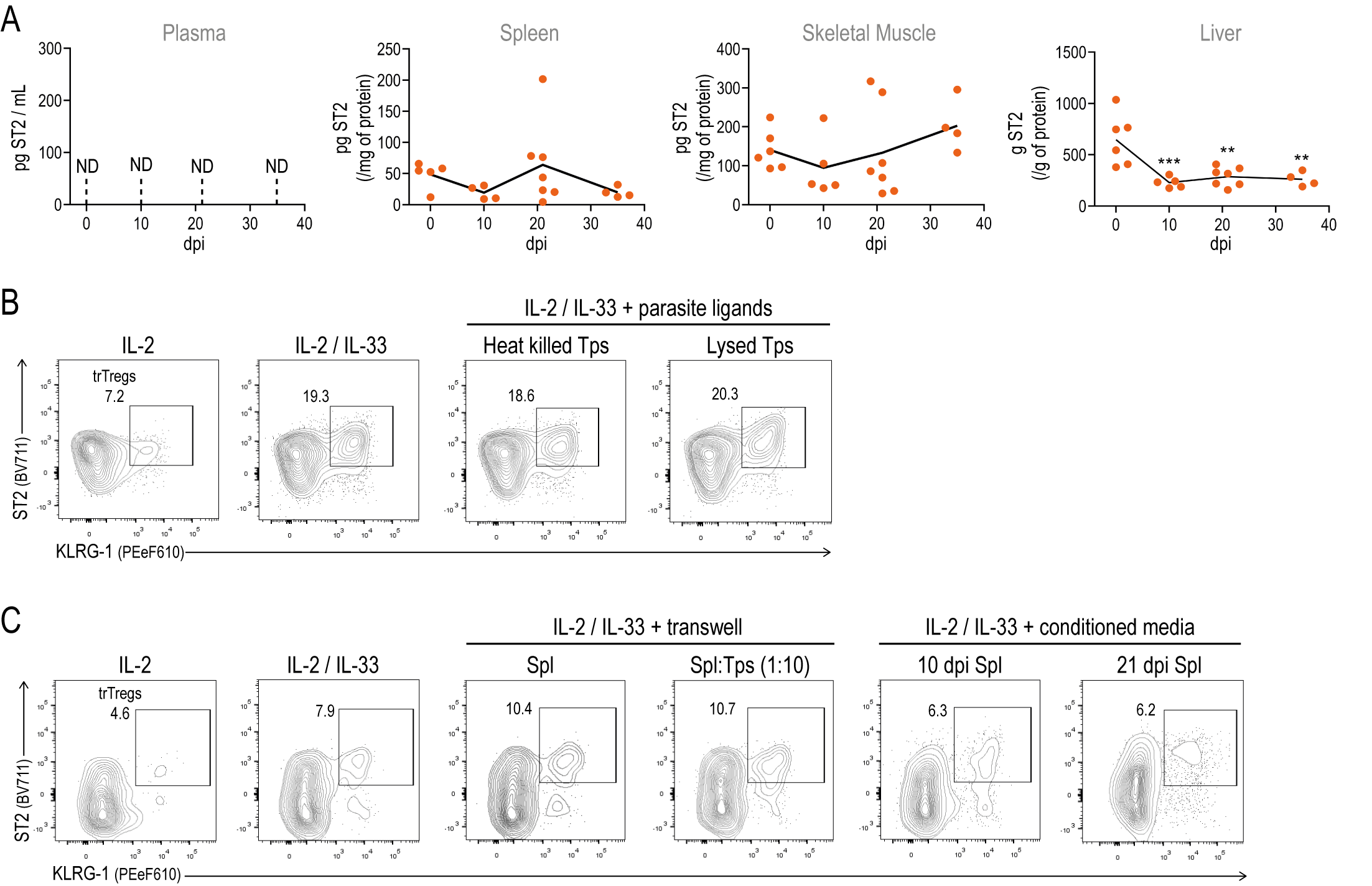

Supplement: S5 Fig — (A) ST2 concentration in plasma, as well as in spleen, skeletal muscle and liver lysates obtained from Foxp3-GFP mice at different days post infection (dpi). Values in tissue lysates were normalized to total protein content and data are presented as individual replicates (circles) and mean (line). Statistical significance was determined by one-way ANOVA. P values are relative to 0 dpi: **p < 0.01; ***p < 0.001. Representative of one experiment. ND: non-detectable. N = 4–13 per group for plasma. (B and C) Representative dot plots showing the frequency of ST2+ KLRG-1+ Tregs (trTregs) within total Tregs isolated from the spleen of non-infected Foxp3-GFP mice and incubated for 72 h with anti-CD3+anti-CD28 together with the addition of different cytokines as follow: IL-2; IL-2+IL-33 and IL2+IL33 plus: microbial ligands (B) or transwell co-cultures or conditioned media providing soluble spleen-derived signals (C), as indicated above each plot. Data were collected from 2 independent experiments. Spl: splenocytes, Tps: trypomastigotes. (TIF) [file ppat.1012906.s005.tif]

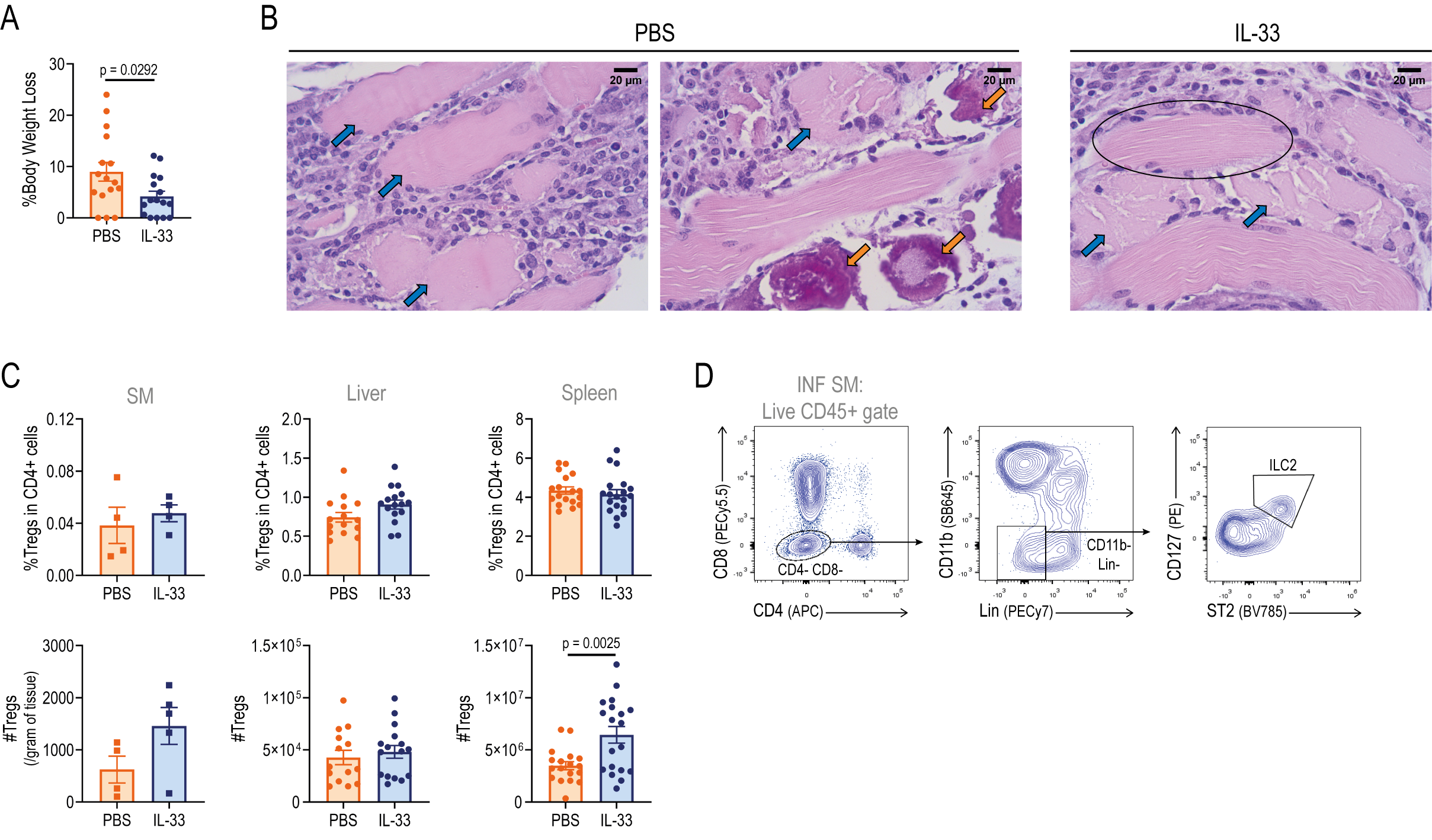

Supplement: S6 Fig — Infected Foxp3-GFP mice received intraperitoneal IL-33 as schematized in Fig 5A. (A) Total body weight loss between 15 and 21 dpi. (B) Representative Hematoxylin-Eosin stain of quadriceps muscle from both groups (as described in Fig 5D) showing histological details. Blue arrows: necrotic muscle fibers, Orange arrows: muscle fiber with dystrophic calcification, circle: preserved skeletal muscle fiber. Magnification: 40X. (C) Bars displaying Tregs frequency within total CD4+ cells (upper row) and absolute number (bottom row) in skeletal muscle (SM), liver and spleen. For SM, squares represent values obtained from pools of 2–4 mice and cell counts are normalized to tissue weight. For liver and spleen, circles represent values from individual mice. (D) Gating strategy used to identify type 2 innate lymphoid cells (ILC2) as CD45+ CD4- CD8- Lin (CD3, CD19, NK1.1, CD11c)- CD11b- CD127+ ST2+ cells. Dot plots are representative of SM from IL-33-treated infected (INF) mice at 21 dpi. (A and C) Statistical significance was determined by unpaired t test. Data are representative of two (A and B) and three (C) independent experiments. (TIF) [file ppat.1012906.s006.tif]

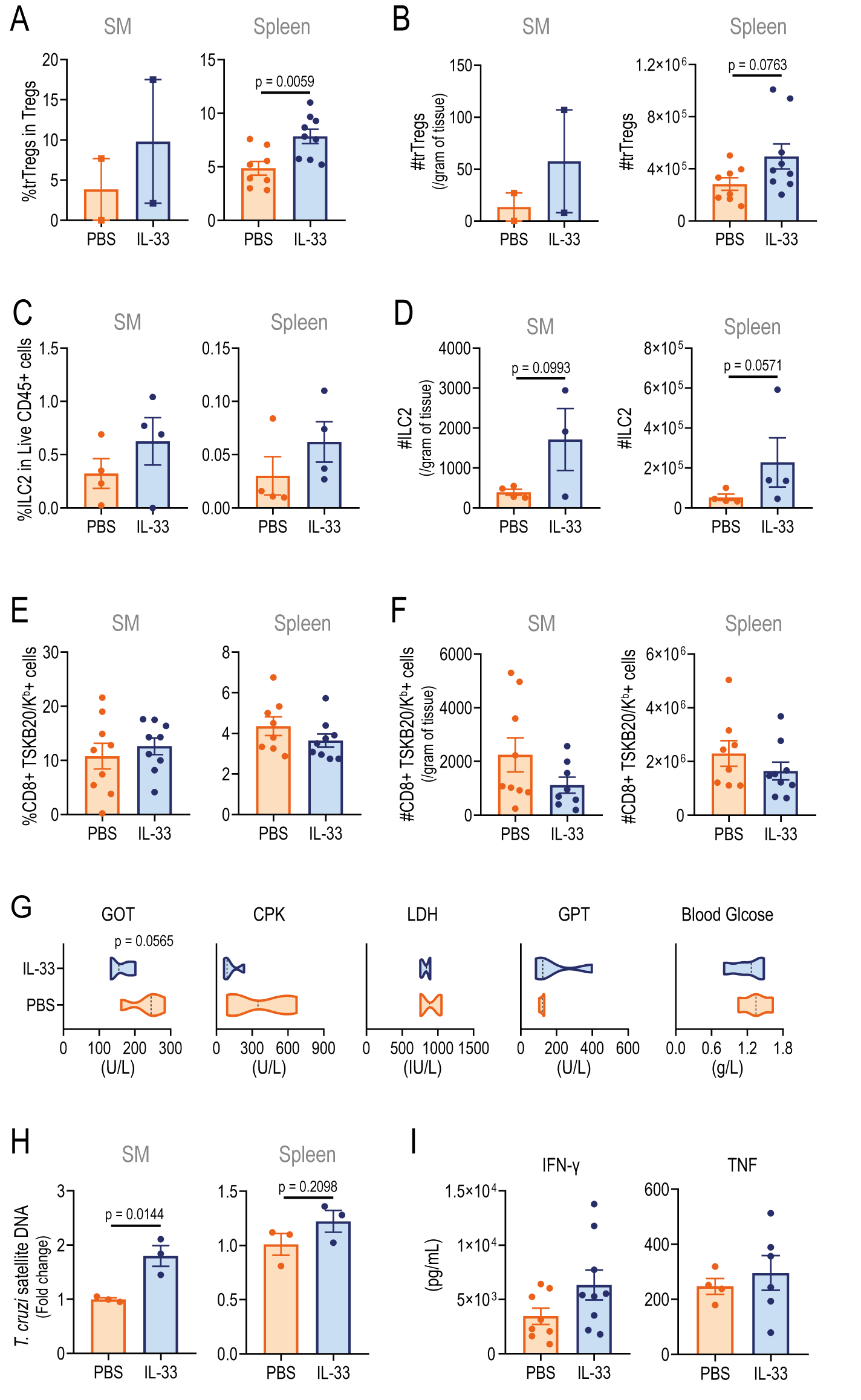

Supplement: S7 Fig — Infected Foxp3-GFP mice received intraperitoneal IL-33 as schematized in Fig 5A and were evaluated at 10 days post infection. (A) Frequency within Tregs and (B) absolute numbers of trTregs in skeletal muscle (SM) and spleen. (C) Frequency within live CD45+ cells and (D) absolute numbers of ILC2 in SM and spleen. (E) Frequency within total CD8+ T cells and (F) absolute numbers of parasite-specific CD8+ T cells in SM and spleen. (G) Violin plots displaying the distribution of plasma GOT, CPK, LDH and GPT activities and glucose concentration. N = 4 per group. (H) T. cruzi satellite DNA quantification in SM and spleen. For each tissue, the graphs represent the fold change in parasitic load in IL-33 treated animals relative to the PBS-treated group. (I) Plasma IFN-γ and TNF concentration. (A-F, H and I) Bars represent the mean ± SEM and circles depict individual replicates. For SM, squares represent values obtained from pools of 2–4 mice (A and B) and cell counts are normalized to tissue weight (B, D and F). Statistical significance was determined as follow: Unpaired t test (A-F, H and I), Mann-Whitney test (G). Data are representative of two (A, B, E, F, H, I) and one (C, D, G) independent experiments. (TIF) [file ppat.1012906.s007.tif]

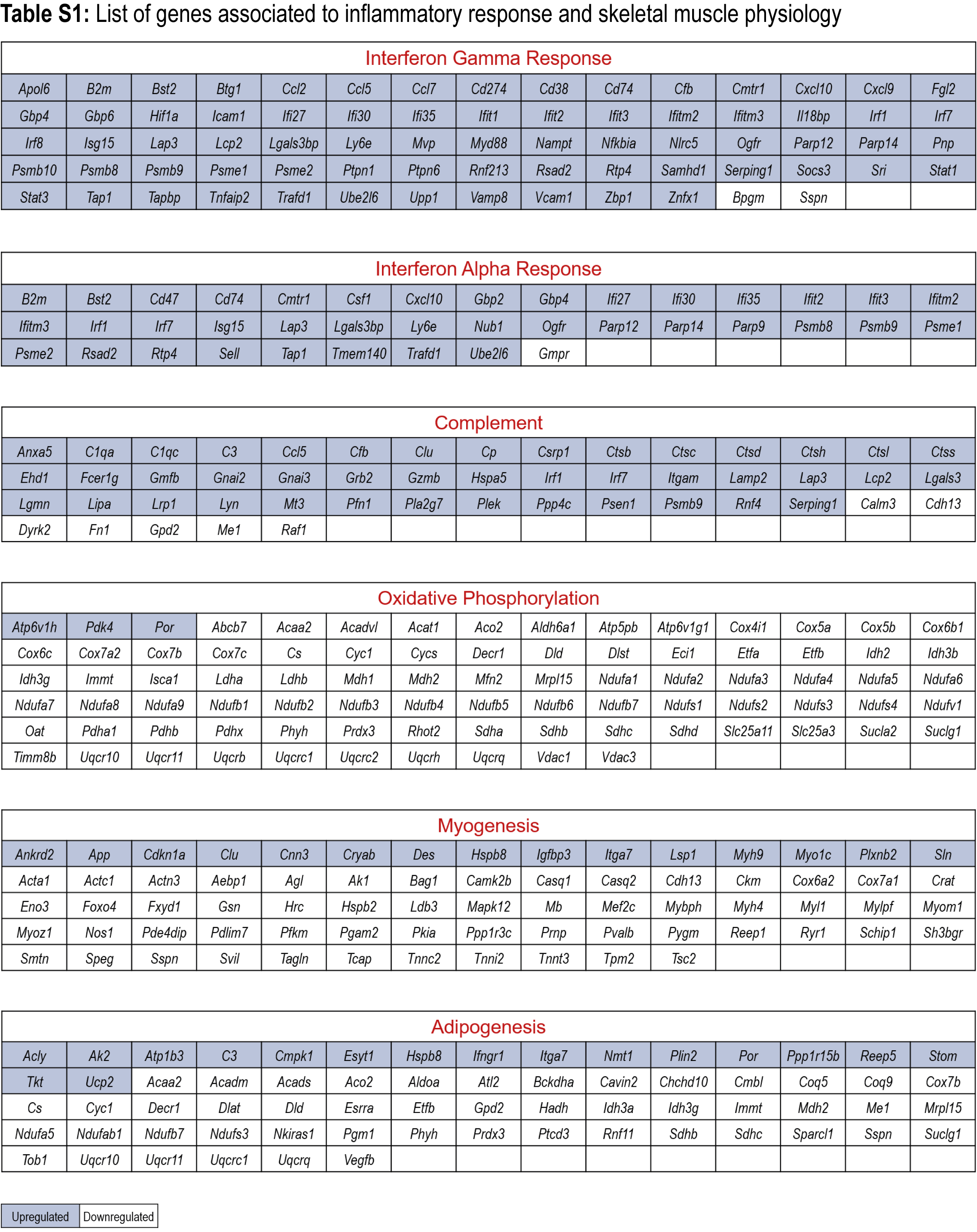

Supplement: S1 Table — Differentially expressed genes between skeletal muscle from infected (15 days post infection) and non-infected mice are shown for each cellular pathway. (TIF) [file ppat.1012906.s008.tif]
