# Supplementary material for: Dynamics of tissue repair regulatory T cells and damage in acute Trypanosoma cruzi infection
Source: PLoS Pathog. 2025 Jan 30;21(1):e1012906. doi: 10.1371/journal.ppat.1012906 (PMC11813105; doi:10.1371/journal.ppat.1012906)
Supplement: Attachment — Submitted filename: Response to reviewers Boccardo et al.docx [file ppat.1012906.s009.docx]

**Answer to reviewers**

"Dynamics of tissue repair regulatory T cells and damage in acute Trypanosoma cruzi infection" (PPATHOGENS-D-24-01779)

**We thank the reviewers for their thorough evaluation of our manuscript and valuable feedback. We have carefully addressed each point raised, performing additional experiments and analyses where possible to strengthen our conclusions. New data have been incorporated into the revised manuscript, while some results are shown exclusively for reviewer reference to avoid overloading readers with information not central to the manuscript’s main conclusions.**

**The findings from early IL-33 treatment are both relevant and meaningful in advancing the understanding of reparative responses in the context of infection. These results underscore the need for future research to investigate the interactions between trTregs, ILC2s, and CD8+ T cells, as well as their individual contributions to infection progression. While these questions will require further extensive and challenging studies, this revised version provides significant insights that are novel and highly relevant to the *PLOS Pathogens* audience. We are confident that the manuscript meets the high standards of quality and interest for the journal.**

Reviewer's Responses to Questions

**Part I - Summary**
 Please use this section to discuss strengths/weaknesses of study, novelty/significance, general execution and scholarship.

Reviewer #1: This paper proposes that tissue repairs Tregs might play a role in the immunopathogenesis of infection by T. cruzi. The approach used in initially descriptive and the authors clearly demonstrate a deficiency of trTregs in site of infection accompanied by a systemic deficiency in IL-33. The authors then attempt to correct the deficiency in IL-33 by administering it starting on day 15 pp.i. but observe no change in the numbers of Treg. They then administer IL-15 at disease initiation and do observe an increase in trTreg and an improvement in survival. However, it is difficult to prove that the beneficial effects of IL-33 are mediated via trTreg as marked increases in ILC2s and antigen-specific CD8+ T cells are also observed.

**Your response is clear and well-structured, but I suggest minor edits to improve flow, avoid repetition, and emphasize the significance of your findings. Here's a revised version:**

**We thank Reviewer #1 for their thoughtful evaluation of our work. As the reviewer notes, our study begins with a descriptive analysis, providing the first characterization of trTregs in the context of T. cruzi infection and associated skeletal muscle damage. This work addresses a critical gap in understanding Treg-mediated tissue repair during infections. Importantly, our study extends beyond descriptive findings by incorporating mechanistic experiments to investigate the modulation of trTregs in a Th1-biased inflammatory environment and to evaluate the effects of early IL-33 treatment on infection progression.**

**As correctly highlighted, the beneficial effects of IL-33 treatment cannot be solely attributed to trTreg modulation, as the treatment also enhances ILC2 and antigen-specific CD8+ T cell responses. This limitation was discussed in the original version of our manuscript and has been further expanded in the revised version (Lines 594-600, clean revised manuscript)). Additionally, we have provided further explanation and preliminary data in the response to reviewers regarding ongoing research aimed at dissecting the specific contributions of each subset. These studies involve complex experimental approaches currently being established in our lab, which fall beyond the scope of the present manuscript.**

**Nonetheless, we believe our findings are novel and significant, offering key insights into trTreg dynamics, the mechanisms regulating their modulation, and the broader impact of IL-33 treatment on *T. cruzi* infection. Furthermore, our results suggest a potential interplay between trTregs, ILC2s, and CD8+ T cells that could be harnessed to optimize therapeutic strategies for improving disease outcomes.**

Reviewer #2: This is a well-written manuscript from Boccardo and colleagues that investigates regulatory T cells (Tregs), and in particular, tissue repair Tregs (trTreg), during the course of T cruzi infection and disease. Using a mouse model of T cruzi infection, they demonstrate that Treg and trTreg abundance decreases in several host tissues at peak parasitemia, including the spleen, liver, and skeletal muscle. Because trTreg are defined based on their expression of ST-2, the IL-33 receptor, they test whether provision of IL-33 can restore trTreg abundance in tissues and improve disease outcomes. They found that early IL-33 treatment increased trTreg abundance and parasite-specific CD8 T cell responses, which correlated with improved disease outcome and lowered parasite burden. Thus, the findings point to a potential therapeutic potential of IL-33 treatment in context of T cruzi infection.

In general, the data support the claims, but there are several places where the rigor of the data could be improved (outlined below). In addition, there are a few key places where further discussion is needed, as noted below. However, overall the study is well-done and the findings are interesting – well done!

**We thank Reviewer #2 for their positive evaluation of our manuscript and for acknowledging the relevance and interest of our findings. We greatly appreciate the constructive comments and suggestions, which have helped us identify areas where the rigor of the data and the discussion could be further improved. We have carefully addressed each of the points raised and believe that these revisions have strengthened the overall quality and clarity of the manuscript.**

Major critiques:

 1. I find it odd that IL-33 supplementation is associated with an increased abundance of trTreg given that there are so few ST-2+ Treg present. The authors should speculate as to how IL-33 is able to increase the % or # of ST-2+ trTregs without the cells expressing the receptor for the cytokine. Presumably this is through an indirect mechanism, or do they propose that this is due to proliferation of a few ST-2+ Tregs or perhaps conversion of conventional CD4 T cells expressing ST-2 into Foxp3+ trTreg?

**We propose that IL-33 supplementation induces the proliferation of ST2+ trTregs, based on previously published studies and our own data. Studies from different labs have shown that IL-33 mediates the expansion of ST2+ Tregs *in vitro* when combined with IL-2 (10.1038/nature13577; https://doi.org/10.4049/jimmunol.1400481). Notably, this last work demonstrated that IL-33 does not induce proliferation in ST2-negative Tregs or non-Tregs under the same *in vitro* conditions. Additionally, IL-33 has been shown to drive ST2+ Treg expansion in various tissue contexts *in vivo* (10.1016/j.cmet.2015.03.005; 10.1016/j.immuni.2016.01.009; 10.1038/ni.3085).**

**Our *in vitro* experiments further support these findings. Using a proof-of-principle approach, we demonstrated that IL-2 combined with IL-33 significantly increased, or showed a clear tendency to increase, the frequency and absolute numbers of trTregs in splenic Treg cultures from both NI and INF mice, despite their differing basal frequencies (Fig 3B; new S4B Fig). This effect was specific to trTregs, as IL-2 + IL-33 did not induce the expansion of ST2+ and/or KLRG-1+ cells within either Foxp3+ cells or conventional CD4 T cell cultures (new S4C Fig, previously S4B Fig, and data not shown). To strengthen these observations, we included additional biological replicates, now presented as new panels in Fig 3B and S4B Fig, providing further evidence of the ability of IL-33 to expand trTreg populations under *in vitro* conditions.**

2. Rigor of the data needs to be improved:
 a. In general, show all the data points rather than just mean and SEM (for example, in Fig 1A-C and 2B). This will help to clarify how many mice are included in each experiment, as it is not always stated in the figure legends.

**Following the reviewer’s suggestion, we have updated most figures to include individual data points rather than showing only the mean and SEM. However, for certain experiments, such as those in new Fig 1B and 1C, we retained the depiction of mean ± SEM, as including individual data points in these cases made the figures visually cluttered and less informative. Additionally, we carefully revised the figure legends to explicitly state the number of mice included in each experiment.**

b. Please show representative flow cytometry dot plots (and not just histograms as shown in Fig S3) for Fig 2C. There are clearly very few trTreg in the spleen (as shown in Fig 2A-B), calling into question the reliability of flow cytometry data looking at activation markers on this very small subset of cells. How many trTreg were used per tissue for this downstream analysis shown in Fig 2C? Showing the numbers of trTreg and KLRG1-ST2- Treg would be informative in this case, so that we can be sure that the % of trTreg expressing all the of markers shown in Fig 2C (as compared to KLRG1-ST2- Treg) are not near zero due simply to the lack of trTreg present in the tissue at 21 days post-infection.

**Following the reviewer’s suggestion, we revised S3 Fig to include representative dot plots for each staining, showing trTregs (ST2+KLRG-1+) and ST2-KLRG-1- Tregs from the spleens of both NI and INF mice. This update allows for a clearer evaluation of differences among cell subsets and a better perception of the number of events analyzed. This reanalysis was performed using an updated version of FlowJo software, resulting in minor differences in MFI values but no changes to the overall conclusions. Additionally, the heat map in Fig 2C has been updated to reflect these adjustments, and a mistake in the subset legends has also been corrected.**

c. There are several places where flow plots are shown without quantification, so it appears that there is only an N of 1 (for example, Fig 3B and Fig 4A). Technical or experimental replicates should be shown with statistical analysis for increased rigor. In many cases, differences that appear very minor are emphasized, and a statistical analysis could perhaps help to back up the author’s claims (for example, differences in Fig 4A are very slight).

**Following the reviewer’s request, we have updated Fig 3B and Fig 4A to include, alongside representative plots, the results of biological replicates for the *in vitro* experiments. These updates allowed us to perform statistical analyses, revealing significant differences in some cases and clear tendencies in others. While the number of replicates was necessarily limited due to the large number of mice required for cell purification in these *in vitro* assays, this proof-of-principle approach is further complemented by in vivo studies. Together, these findings strengthen our conclusions and ensure greater rigor in data presentation.**

3. Survival experiments need to be compared between figures and the differences in control groups need to be accounted for. In figure 4C, the control group has 100% survival, whereas in figure 5B, it looks as though apx 50% of the control mice succumb to infection. This is important because it calls into question the significance of the findings. Are there differences in the control groups between the two experiments that could account for these major differences? One way to reconcile this would be to show survival curves and % change in body weight (relative to day 0) following infection in Figure 1 to establish the model.

**We appreciate the reviewer pointing out the apparent differences in survival between Fig 4C and Fig 5B, which provided us with the opportunity to clarify our model. In our infection model, 40–50% mortality occurs within a narrow window during the acute phase of infection, typically between days 20–22 and 35–38 post-infection, coinciding with high parasitemia and weight loss. Beyond this period, all surviving mice remain alive for at least 350 days (data not shown).**

**The observed differences in survival between Fig 4C and Fig 5B likely reflect expected variability in mortality timing, further influenced by differences in experimental endpoints. Specifically, Fig 4C shows an early endpoint at 21 dpi, while Fig 5B extends to 35 dpi and now up to 60 dpi in the revised figure.**

**To address the reviewer’s suggestion and reconcile these differences, we have included survival curves and body weight loss curves relative to day 0 in Fig 1A and S1A Fig, establishing the dynamics of infection progression and survival in our model.**

4. The authors show in Fig 5 that provision of IL-33 early after infection results in an increased frequency of Ag-specific CD8 T cells in the SM, liver, and spleen (Fig 5I), yet no difference in plasma IFNg and a decrease in plasma TNFa (Fig 5L). To see if there is a functional difference in the CD8 T cells, it could be useful to perform intracellular cytokine staining to look at cytokine expression on a single-cell level, or perhaps measure tissue lysates for cytokines. Alternatively, can the authors speculate on how IL-33 supplementation might be reducing the parasite burden (Fig 5K)?

**CD8+ T cell responses during *T. cruzi* infection have been shown by us and others to correlate with tissue parasitism control (10.1371/journal.ppat.1007410; 10.1016/j.pt.2019.08.006). In addition, cytotoxic effector molecules have been demonstrated to play a role in CD8+ T cell-mediated protection (10.1006/expr.2000.4498; 10.1002/immu.200390009). Therefore, we associate the reduced parasite burden observed after IL-33 treatment with the increase in parasite-specific CD8+ T cells, despite IL-33 not affecting systemic IFN-γ levels and even reducing TNF levels.**

**In the original version of the manuscript, we chose not to include additional results evaluating CD8+ T cell functionality, as they did not provide further support to our conclusions and might divert the readers' attention. Results obtained by intracellular cytokine staining of CD8+ T cells from the spleens of IL-33-treated and control mice demonstrated that IL-33 supplementation did not alter CD8+ T cell functionality. Specifically, CD8+ T cells from both groups exhibited similar frequencies of IFN-γ, TNF, and Granzyme B expression upon PMA/Ionomycin stimulation (see figure below for reviewer reference). These findings suggest that IL-33 enhances the magnitude of CD8+ T cell immunity by increasing the frequency of parasite-specific CD8+ T cells rather than improving their quality, as defined by effector molecule expression at the single-cell level.**

**We have revised the text to emphasize our interpretation that the observed reduction in parasite burden following IL-33 treatment is primarily attributed to the increased frequency of parasite-specific CD8+ T cells (Lines 433–436, clean revised manuscript). However, we still believe that including this additional data would unnecessarily detract from the manuscript's focus without altering the main conclusions.**

5. It would be nice for the authors to include some histology data to show a decrease in tissue pathology associated with an increase in trTreg abundance following IL-33 treatment. This would firmly establish that trTreg are indeed “tissue repair” Tregs beyond use of phenotypic markers.

**Following the reviewer’s suggestion, we performed a histological analysis of SM from IL-33-treated and control mice. Our results revealed a clear reduction in tissue damage in IL-33-treated mice, as evidenced by milder necrosis and dystrophic calcification compared to the control group. Specifically, histological examination of the control group showed severe necrosis and moderate to severe dystrophic calcifications in ~70% of the samples, accompanied by predominantly multifocal mononuclear inflammatory infiltrates in ~85% of the samples. In contrast, IL-33 administration improved histological parameters, with severe or moderate necrosis and dystrophic calcification reduced to ~30% of the samples, with no changes in infiltration.**

**This reduction in pathology correlated with the observed increase in trTregs following IL-33 treatment, supporting their potential role in tissue repair. Representative histological images and quantification graphs have been included as a new Fig 5D. These findings have also been incorporated into the Results section (Lines 391–399, clean revised manuscript) and briefly discussed in the revised Discussion (Lines 549–551, clean revised manuscript).**

6. It would be interesting to try i.m. IL-33 supplementation early after infection or perhaps even pre-infection to see if that has a stronger effect on trTreg in skeletal muscle. However, I do not consider that experiment to be essential for publication of this manuscript. Or was IL-33 delivered i.m. in the experiment shown in Figure 5 – the delivery route was not clear from the figure legend.

**The reviewer’s comment prompted us to clarify in the revised figure legend and experimental scheme that IL-33 treatment in Fig 5 was administered systemically via i.p. injection. While we agree that early i.m. IL-33 administration might have a stronger effect on trTregs in SM—supported by our findings in NI mice treated with i.m. IL-33—exploring both systemic and local routes would have significantly increased the number of mice used in this study. We opted for systemic administration considering evidence that trTreg differentiation occurs in sequential steps, with initial commitment taking place in secondary lymphoid organs. Systemic delivery of IL-33 likely ensures broader accessibility to these organs, promoting the development or expansion of committed trTreg precursors, which subsequently migrate to peripheral tissues, including SM. This approach allowed us to evaluate IL-33-mediated effects on tissue repair and damage mitigation across multiple organs.**

Minor critiques:

 1. In the introduction, the authors refer to trTreg in many places where the citing literature was studying tissue Treg. Please take care to clearly distinguish analyses of tissue Tregs in general from trTreg (as not all tissue Tregs have a trTreg phenotype). As an example, in line 66-69, the statement seems to refer more broadly to tissue Treg, although the authors attribute the statement to trTreg. There is a similar problem with lines 72-74.

**We thank the reviewer for pointing out this important issue. It is indeed a common mistake to confuse tissue Tregs, which encompass all Tregs residing in peripheral tissues, with trTregs, a specialized subset of Tregs dedicated to tissue repair. While some reviews use the terms interchangeably, this is not accurate. Upon reviewing our introduction, we agree with the reviewer that these terms were occasionally mixed, contributing to potential confusion. To address this, we have extensively revised the second paragraph of the introduction to clearly differentiate between tissue Tregs and trTregs. Additionally, the entire manuscript has been thoroughly reviewed and revised to ensure consistent and accurate use of these terms throughout.**

2. There is a typo in lines 207-209 – the data in Fig 2C do not show that spleen trTreg from non-infected mice exhibit higher expression levels of most markers compared to ST2-KLRG1- Treg but rather the opposite.

**Upon reviewing the comment, we realized that there was a mistake in the legend for Fig 2C, as the labels were inverted. This error has been corrected in the revised Fig 2C. Additionally, following reviewer’s suggestion, we have replaced the histograms in S3 Fig with representative dot plots, providing a clearer visualization of the expression differences.**

3. The sentence in lines 220-222 is unclear; in particular, the phrase “presenting particularities likely as a consequence of the infection”. Please revise to clarify what you mean. Furthermore, please clarify what you mean by “bone fide trTreg”.

**Following the reviewer’s suggestion, we have clarified that *bona fide* trTregs are defined based on the phenotype described by Delacher et al. (doi:10.1038/ni.3799). Additionally, we have revised the text between lines 216–241 to improve clarity and better describe the results. The revised text emphasizes the main conclusions: trTregs from both NI and INF mice exhibit higher expression of trTreg-associated markers compared to their ST2-KLRG-1- Treg counterparts. Furthermore, when comparing trTregs from INF mice to those from NI mice, the former display higher expression levels of many markers analyzed, likely influenced by the infection.**

4. Line 250: please fix typo “Ssplenic”.

**The typo has been corrected in the revised manuscript.**

5. In Figures 1C and 1E, please clarify in the figure legend how the number of CD45+ cells in tissue was quantified (was this flow cytometry, or microscopy, or something else?).

**Based on the reviewer’s comment, we realized that the axis labels in Fig 1C and 1E were misleading. These figures represent the total cell counts of splenic cells and SM-infiltrating leukocytes, respectively, as determined using a Neubauer hemocytometer after preparing cell suspensions, as described in the Materials and Methods. The axis label for Fig 1C has been corrected to "Total splenocyte count" and for Fig 1E, it now reads "Leukocytes/gram of tissue" reflecting cells obtained following a Percoll gradient of SM cell suspensions. Similar corrections have been made in Fig 4E and S1D Fig.**

6. Please indicate the limit of detection for IL-33 detection in Fig 3A.

**The limit of detection for IL-33 quantification has been added as a dashed grey line in the graph of plasmatic IL-33 concentration in Fig 3A, with the reference included in the figure legend.**

7. In Fig S4B there appears to be a typo – I believe this is looking at CD4+Foxp3GFP-negative cells, yet the figure indicates “Tregs gate”.

**We thank the reviewer for pointing out this mistake, which has been corrected in the revised figure.**

Reviewer #3: The data presented are indeed relevant within the context of acute T. cruzi infection in the animal model. However, the results appear somewhat controversial compared to previous findings from the same group regarding the role of conventional Treg cells. In their earlier work, it was observed that depletion of this Treg population led to an increase in parasite-specific CD8+ T cells (as measured using e TSKB20 and TS peptide) and a decrease in parasitemia.

In the current study, while IL-33 is shown to promote the expansion and activation of regulatory T cells, you report an increase in the same population of parasite-specific CD8+ T cells alongside a decrease in parasite load in tissues, which presents opposing effects. Although you have made attempts to explain these controversial results, further elaboration may be necessary to elucidate a possible physiological mechanism.

**We thank the reviewer for their positive evaluation of the relevance of our current work. We are convinced that the results presented in this manuscript are not controversial with respect to our previous report, as they can be reconciled by considering the distinct roles of specialized Treg cell subsets. As noted, our current study builds upon earlier findings from our laboratory, which demonstrated that during the acute phase of *T. cruzi* infection, Tregs become activated and acquire Th1 specialization features. These activated Tregs exhibit enhanced regulatory capacity, suppressing effector CD8+ T cell responses through CD39-mediated mechanisms (10.3389/fimmu.2018.02555; 10.1371/journal.ppat.1012191). Consequently, the systemic depletion of total Tregs in infected DEREG mice enhanced CD8+ T cell immunity and parasite control.**

**In this manuscript, we focused on a distinct specialized Treg subset, trTregs, characterized by ST2 and KLRG-1 co-expression, which are expanded following IL-33 treatment both *in vitro* and *in vivo*. While trTregs are known to sustain stromal cell proliferation and growth (10.1084/jem.20231244), their specific role in regulating CD8+ T cells has not been directly explored. Although Treg cells are traditionally associated with limiting anti-pathogen immunity, early reports demonstrated that Tregs can support the proper recruitment of protective CD8+ T cells into tissues and contribute to the development of local immunity in certain infections (10.1126/science.1155209; 10.1128/jvi.00036-09; 10.4049/jimmunol.1000423). More recently, a specialized local Treg population has been shown to sustain tissue-resident CD8+ T cell responses critical for controlling the protozoan parasite *Eimeria vermiformis* (10.1038/s41590-020-0674-9).**

**Thus, our findings can be understood by considering that specialized Treg subsets have diverse, context-dependent functions. The trTregs expanded by IL-33 supplementation early during infection may not efficiently suppress CD8+ T cell responses but may act primarily on tissue stromal cells to promote repair. Together, these results suggest that IL-33 treatment fosters an environment where trTregs contribute to tissue repair, while their potential suppressive effects on CD8+ T cells may be negligible. This could stem from IL-33 directly promoting antiparasitic CD8+ T cell responses, as previously reported (10.1126/science.1215418; 10.1016/j.immuni.2023.01.029), or indirectly through other IL-33-responsive subsets, such as ILC2, which have been shown to promote CD8+ T cell immunity (10.1158/2159-8290.CD-RW2020-032; 10.1080/2162402X.2023.2243112; 10.1016/j.bbrc.2022.11.006).**

**Collectively, our results highlight early IL-33 treatment during acute *T. cruzi* infection as an interesting model to study how robust effector immune responses and tissue repair-specialized regulatory responses can coexist and synergize to improve infection outcomes. Further research in our laboratory will aim to delineate the individual contributions of the immune cell populations targeted by IL-33 in the observed positive effects on infection progression.**

**To address the reviewer’s request for further elaboration, we have expanded and refined the discussion to emphasize the distinct regulatory functions of Treg subsets and the potential direct role of IL-33 on CD8+ T cells (Lines 576-593, clean revised manuscript).**

The data presented in Figure 2 are expressed as mean ± SEM. However, I would like to clarify the methodology regarding the normalization of cell counts for the SM tissue. The text states that cell counts are normalized to tissue weight and that all values were obtained from pools of three mice, with N = 2-7 per day post-infection (dpi). If there were only two mice per dpi, could you please elaborate on how a pool of three mice was achieved?

**We thank the reviewer for pointing out that the figure legend may have been unclear. For the evaluation of SM cell populations, particularly trTregs, cell suspensions were obtained from pools of 2 to 4 mice, with cell counts normalized to tissue weight. From these pools, data from 2 to 7 pools per dpi were plotted, which correspond to 4 to 28 mice, respectively. Additionally, for subsets present at higher frequencies in tissues such as the spleen and liver, data from individual mice were used.**

**In response to Reviewer #2’s suggestion, we revised the figures to present most of the data as individual values, with pools represented by square symbols and individual mice by circles. Specifically, Fig 2B now includes a larger number of values, with N = 4–7 SM pools (squares) and N = 4–16 individual spleen or liver samples (circles) per dpi. These changes improve the clarity of the data presentation while maintaining consistency across figures. Legend for figures were updated accordingly.**

Regarding Figure 3D, it appears to depict flow cytometry results for trTreg within Treg populations. However, the description as "Flow cytometry analysis of Tregs present in..." may lead to some confusion, particularly since no frequency or parameters for this population are calculated. I suggest rephrasing this section for clarity.

**We appreciate the reviewer’s observation about the legend for Fig 3D. It has been rephrased for clarity to avoid potential confusion. It now reads: "D) Dot plots depicting the frequency of trTregs within total Tregs in the spleen of non-infected (NI) Foxp3-GFP mice 72 hours after receiving 3 doses of intraperitoneal IL-33 or PBS, as described in (C)."**

Additionally, it may be beneficial to include data corresponding to non-infected (NI) animals in a manner similar to that of infected mice, rather than presenting it as a line of media value. This could provide more context for the findings.

**Following the reviewer’s suggestion, we have included data corresponding to NI animals in Fig 3E, presented in the same manner as for infected mice. This addition provides better context for the findings.**

It is somewhat unclear why both individual samples and pooled samples were used for the same tissue type, such as SM. Could you provide insight into this methodological choice?

**Given the low frequency of trTregs and the small number of cells present in tissues like SM, we used pools from different mice in experiments measuring trTreg frequency and numbers in SM (Fig 2B, Fig 3E, Fig 5E-F, S7A-B Fig). Exceptionally, we also used pools of SM and heart in one experiment to determine total Tregs, due to the similarly low cell yields in these tissues (S2B and S6C Figs).**

**Additionally, following the reviewer’s comment, we identified an error in one of the figures (Fig 3E) regarding the symbols used to represent pooled versus individual values. In the revised manuscript, we have thoroughly reviewed all figures to ensure consistency and clarified the methodological choices in the Materials and Methods section (Lines 676–678, clean revised manuscript).**

I would appreciate some clarification on why it was not feasible to dilute the liver sample to fall within the assay's dynamic range. Understanding this limitation would be helpful.

**To bring the quantification of sST2 in liver lysates within the assay’s dynamic range, a very high dilution was required, which caused significant variability likely due to matrix effects. However, during the revision, we optimized the assay to achieve good reproducibility even at high dilutions. As shown in the revised S5A Fig, sST2 concentration is markedly higher in the liver compared to other tissues and is downregulated during infection. These results have been incorporated into the Results section (lines 326-327, clean revised manuscript).**

Parasite Load Measurement

 Did you measure the parasite load in mice treated with IL-33 alone, as well as in those treated with both IL-33 and IL-2?

**In our initial approach, where IL-33 alone or IL-33+IL-2 was supplemented from day 12 post-infection, we did not measure parasite load because our data showed no significant changes in CD8+ T cell responses under these conditions, as depicted in the figure provided below for the reviewer’s reference. Thus, we considered there was no rationale to justify further parasite load quantification. Since these results do not directly contribute to the main message of our study, we opted not to include additional graphs and results that could divert from the focus of the manuscript.**

**As outlined in the original manuscript, tissue parasite load for the early IL-33 treatment experiments was measured at both 10 dpi and 21 dpi. These results are also presented in the revised manuscript as Fig 5K and S7H Fig.**

Furthermore, do you have data on mice treated with IL-33 and IFN-gamma? This information would be valuable to determine if there is a direct correlation between the TH1-like response and the effect of IL-33 on rtTreg.

**We appreciate the reviewer’s insightful comment and the opportunity to clarify our approach. We have not tested *in vivo* experimental groups where mice were treated with IL-33 in combination with IFN-γ supplementation, as *T. cruzi* infection naturally induces robust IFN-γ production. Instead, as presented in the original manuscript we performed experiments where IFN-γ was neutralized to investigate trTreg dynamics under restrained Th1 responses (Fig 4), without additional manipulation of IL-33.**

**While combining IFN-γ neutralization with IL-33 supplementation could be an interesting approach, our results demonstrated that IFN-γ neutralization partially compromised parasite control (Fig 4D). Based on these findings, we hypothesized that additional IL-33 supplementation in this immunodeficient context might exacerbate infection and mortality by enhancing Treg cell immunosuppressive functions, as previously described (10.4049/jimmunol.1400481). It is worth noting that Matta et al. observed enhanced immunosuppression *in vitro* for both ST2+ and ST2- Tregs from IL-33-treated mice. However, the limitations of classical *in vitro* approaches for studying tissue Treg biology have been highlighted by experts, who advocate for the use of more relevant *in vivo* models (10.1038/s41577-021-00519-w).**

**To mitigate potential complications from increased vulnerability to infection during a well-established *T. cruzi* infection, we adopted an alternative experimental approach based on the positive results of IL-33 supplementation in NI mice. Specifically, we administered IL-33 at early time points during infection, before the IFN-γ-dominated Th1 response was fully established. This strategy allowed us to evaluate IL-33 effects under conditions of low IFN-γ levels while minimizing risks to mouse survival and optimizing the ethical use of animals.**

**Using this early IL-33 supplementation strategy, we observed a significant increase in trTregs, as intended. Additionally, IL-33 treatment was associated with an increase in ILC2 numbers and enhanced CD8+ T cell immunity, both effects consistent with previous reports (10.1038/s41467-019-10733-9; 10.3389/fimmu.2021.669787; 10.1002/eji.201948413; 10.1126/science.1215418). Collectively, these IL-33-induced effects correlated with reduced tissue damage and improved parasite control, ultimately resulting in milder infection progression. Notably, these findings highlight the complexity of *in vivo* immune crosstalk, which may not always be fully anticipated by *in vitro* experiments.**

did you determine the frequency of Treg cells across the different treatment groups? This information would enhance our understanding of the immune response in your study, and is in relation with my previous argumentation.

**We have data on the frequency and absolute numbers of total Tregs across the different treatment groups. In the original version of our manuscript, we chose to exclude these data to streamline the figures and focus on the most relevant findings. However, considering the reviewer’s suggestion, we have reconsidered presenting some of this information.**

**For the IL-33 and IL-2/IL-33 supplementation experiments described in Fig 3, IL-33 treatment did not increase Treg frequency or absolute numbers in any of the tissues evaluated. Conversely, IL-2/IL-33 treatment led to a reduction in Treg percentages and absolute numbers in the spleen and liver (see figure provided below for reviewer reference). As these findings are not directly relevant to the manuscript's main conclusions, we opted to exclude them to preserve focus and clarity.**

**In contrast, in the experimental approach described in Fig 5, early IL-33 treatment had a variable effect on the total Treg pool: no changes in frequency in SM, liver, or spleen, with significant increases in absolute numbers observed only in the spleen (now shown in S6C Fig). However, IL-33 supplementation consistently expanded trTregs across all evaluated tissues (Figs 5E and 5F).**

**Altogether, these findings suggest that the effect of IL-33 on the total Treg pool depends on the frequency of ST2-expressing cells capable of responding to this cytokine, with trTregs being among the subsets increased. Consequently, the role of this specialized subset in tissue repair is particularly enhanced by IL-33 treatment. To strengthen the conclusion regarding the positive effect of IL-33 treatment on tissue damage, specifically linked to trTregs expansion, we have incorporated new histological data into the revised manuscript. These results demonstrate reduced SM damage in IL-33-treated mice (new Fig 5D and S6B Fig). These results have been integrated into the Results and Discussion sections of the revised manuscript (Lines 391–399 and 549–551, clean revised manuscript).**

**Part II – Major Issues: Key Experiments Required for Acceptance**
Please use this section to detail the key new experiments or modifications of existing experiments that should be absolutely required to validate study conclusions.

Generally, there should be no more than 3 such required experiments or major modifications for a "Major Revision" recommendation. If more than 3 experiments are necessary to validate the study conclusions, then you are encouraged to recommend "Reject".

Reviewer #1: The authors clearly show a diminution in trTreg at the sites of infection and a diminution in systemic IL-33. This finding appears solid but the never directly demonstrate that the two observations are mechanistically related. There are several areas of the manuscript that need to be addressed:
 1. The authors contend that the trTreg from infected mice are intrinsically capable of responding to IL-33 based on their capacity to expand in vitro in response to anti-CD3/CD28/IL-2/IL-33 administration (fig. 3B). This study is performed starting with a total Treg population. The authors need to show absolute counts of Total Treg and trTreg at the end of the culture to prove actual expansion. It is also very difficult to conclude that this in vitro study using maximal stimulatory conditions has any relevance to the lack of response seen in vivo.

**Following the reviewer’s suggestions, we increased the number of replicates in the *in vitro* expansion assays to N=4-6 (new Fig 3B). We consider this number of replicates to provide robust statistical significance while balancing ethical considerations, given the high number of mice required for these experiments. Sorting Tregs from INF mice, where they are present at low frequency, necessitates the use of a significant number of animals. Statistical analyses now show that the presence of IL-33 in Treg cultures, both in NI and INF Tregs cultures, leads to an increase or clear tendency for increased frequency and/or absolute cell counts of trTregs, but not total Tregs (new S4B Fig).**

**Although we used culture conditions described in the literature (10.1371/journal.ppat.1007410; 10.1016/j.pt.2019.08.006), we agree that *in vitro* experiments under specific stimulatory conditions cannot be directly extrapolated to *in vivo* scenarios. Instead, these assays were intended as a proof of principle. To further validate our findings, we performed additional *in vivo* experiments. We believe that the combined use of *in vitro* and *in vivo* approaches provides robust support for our conclusions. We have revised the manuscript to include a brief discussion of the limitations of these approaches (Lines 526-529, clean revised manuscript).**

2. The authors then postulate that the presence of IFNg is responsible for the failure of the trTregs to expand in vivo. The experiment shown in fig. 4A is far from convincing. It is again based on the in vitro assay and the purported suppression of expansion (7.9-6.1%) is surely not suppressive. This is a single experiment without statistical variation. The authors show that TNF appears to play a beneficial effect in survival, but the studies with anti-IFNg are not carried out long enough to observe changes from controls (cf.fig. 4c and 5b). The claimed increases in the percentages and absolute numbers of trTreg after IFNg treatment are very small and likely to mediate any beneficial effects.

**To increase the rigor of the *in vitro* results, we have now included additional replicates and statistical evaluations. As discussed earlier, the *in vitro* experiments were complemented with *in vivo* neutralization studies to provide a broader understanding. It is important to clarify that the aim of these experiments was not to assess survival outcomes, but rather to analyze the effects of Th1 cytokines, specifically IFN-γ and TNF, on trTreg dynamics. The critical role of both TNF and IFN-γ in host resistance during *T. cruzi* infection is well established (10.1128/iai.63.12.4862-4867.1995; 10.1006/expr.1996.0109; 10.4049/jimmunol.166.7.4596). Thus, we chose an early endpoint at day 21 post-infection to focus on trTreg frequencies and avoid confounding effects of increased mortality due to uncontrolled parasite replication caused by cytokine neutralization. Indeed, TNF neutralization caused early mortality, making it unsuitable to evaluate the role of TNF, while IFN-γ neutralization, as shown in Fig 4D, significantly increased parasitemia.**

**Furthermore, we agree with the reviewer that the observed increase in trTregs after IFN-γ neutralization is small, though statistically significant. We do not propose that these changes in trTregs mediate any beneficial effects on infection progression. Instead, they offer insight into a possible mechanism underlying the reduction of trTregs observed during Th1-mediated inflammation. We have introduced a discussion of these aspects in the Results section (Lines 371–376, clean revised manuscript).**

3. The studies on the early administration of IL-33 which do show a beneficial effect using multiple parameters are impressive but certainly do not prove that the effects are secondary to the increase in trTreg. As the authors point out an early increase in ILC2s also occurs and there are likely many more ILC2s than trTreg in vivo. The only way to dissect the contribution of ILC2s is to delete them for example by using GATAfl/flXKLG1 mice or other strains deficient in ILC2s. The contribution of CD8+ T cells could easily be tested by using depleting antibodies a widely accepted technique.

**We agree with the reviewer that the beneficial effects observed with early IL-33 treatment cannot be exclusively attributed to the increase in trTregs, as ILC2s are also expanded, and CD8+ T cell immunity is enhanced. These limitations have been addressed in the manuscript, and we have revised the text to ensure they are clearly presented. We emphasize that our conclusions focus on trTreg dynamics during infection, including the restrictive role of Th1 cytokines in trTreg accumulation, and how IL-33 treatment simultaneously expands trTregs and ILC2s without compromising CD8+ T cell immunity. These immune changes correlate with reduced tissue damage, supported by biochemical markers and newly added histological evaluation of skeletal muscle, as well as decreased parasite replication, ultimately leading to milder infection progression. We believe these findings are novel and of significant interest to the *PLOS Pathogens* audience.**

**The striking phenotype observed with IL-33 treatment opens avenues for future research into the interactions among the affected immune populations and their contributions to infection progression during both acute and chronic stages. However, addressing these questions requires complex experimental approaches, including mouse models such as ILC2 KO, CD8 KO, DEREG, and ST2 KO mice. These models require significant time to import and establish, as in our case with the recently obtained ST2 KO mice for colony development. We have also been working with ILC2 experts to identify suitable ILC2-deficient models. For example, Klrg1CreGata3fl/fl mice were deemed unsuitable as they affect trTregs and CD8+ T cells (10.1016/j.celrep.2023.112073). Instead, we are in the process of importing the R5-IL5;dtTomato-IRESCreLSLRosa26DTA model developed by Dr. Locksley (UCSF) (10.1038/nature12526), though this remains a time-intensive process.**

**Additionally, we have begun exploring the role of CD8+ T cells using CD8α-deficient mice (see Figure below for Reviewer reference). As expected (10.1016/j.pt.2019.08.006), these mice are highly susceptible to *T. cruzi* infection, limiting the experimental timeframe due to early mortality. Preliminary data showed increased mortality in IL-33-treated CD8α-deficient mice. While parasitemia levels were similar between surviving IL-33-treated and control groups, differences in tissue parasitism—which relies more on CD8+ T cell immunity—remain to be assessed. Notably, surviving IL-33-treated mice exhibited a trend toward reduced tissue damage, correlating with a tendency to increased ILC2 and trTregs. However, these preliminary results require further validation, which may take considerable time, and are beyond the scope of the current study.**

**In conclusion, while we acknowledge the importance of dissecting the individual contributions of trTregs, ILC2s, and CD8+ T cells, we believe the current results provide valuable insights into trTreg dynamics, tissue damage, and the immunomodulatory effects of IL-33 during *T. cruzi* infection. We consider these findings to align with the scope and quality of publications in *PLOS Pathogens.***

Reviewer #2: See above

Reviewer #3: (No Response)

**Part III – Minor Issues: Editorial and Data Presentation Modifications**
 Please use this section for editorial suggestions as well as relatively minor modifications of existing data that would enhance clarity.

Reviewer #1: Nonew

Reviewer #2: See above

Reviewer #3: Minor details,

 Line 250: Ssplenic, erase S
 Line 1231, missed space
 Figure S1: missed u in Glucose

**We thank the reviewer for identifying these typos, which have been corrected in the revised manuscript.**
